# Parathyroid autotransplantation during thyroidectomy among the European Society of Endocrine Surgeons: we still do not agree

**DOI:** 10.1007/s13304-025-02510-9

**Published:** 2026-03-17

**Authors:** Muharrem Oner, Maite de Miguel-Palacio, Eduardo Luque-Villalobos, Juan J. Sancho-Insenser, Leyre Lorente-Poch

**Affiliations:** 1 Endocrine Surgery Unit, Department of General and Digestive Surgery, Hospital del Mar, Barcelona, Spain; 2 Department of Surgery, Antalya City Hospital Antalya, Antalya, Turkey; 3 Department of Surgery, Universidad Autònoma de Barcelona, Barcelona, Spain; 4 Department de Medicine and Life Sciences, Pompeu Fabra University, Barcelona, Spain; 5 Hospital del Mar Research Institute (HMRIB), Barcelona, Spain

**Keywords:** Thyroidectomy, Hypoparathyroidism, Survey, Autotransplantation, Parathyroid, Surgical technique

## Abstract

The benefits of parathyroid autotransplantation (PAT) to prevent hypoparathyroidism are controversial. Our aim was to assess current indications and techniques for PAT during thyroidectomy among European Society of Endocrine Surgeons (ESES) members, incorporating the indications derived from indocyanine green (ICG) use, and compare them to a previous survey. An online survey was sent to all ESES members. Answers from 146 members from 30 countries and 132 centers were analyzed and compared to a previous (2015) survey. The commonest indications were “complete-devascularization” (90%) or “recovered-from-specimen” (72%) whereas “difficult-anatomical-disposition” (24%), “color-changes” (17%), and “score 0-ICG” (11%) were less common. The majority of polled surgeons (85%) performed autotransplantation in less than 10% of total thyroidectomies. Timing for autotransplantation varied from “at-the-end-of-the-procedure” (56%), after completing the lobectomy (25%) to immediately (18%). Parathyroids were usually kept in saline (67%) although a moistened gauze or frozen surface were also used. “Chopping” was the preferred fragmentation method (66%), followed by “slicing” (19%) and “triturating” (12%). The majority of surgeons fragmented the parathyroids from 3 mm to non-identifiable sizes and placed the fragments in a single pocket (70%). The number of fragments per pocket varied widely and the most common site for placement was the sternocleidomastoid muscle (90%). Among surgeons who responded to the previous survey, indications and technique did not show major differences, but 15% of them added ICG as a new criterion. The indications and surgical technique for PAT among ESES members were exceedingly diverse. Technical disparity may play a role in controversial results. Standardization initiatives are warranted.

**Supplementary Information:**

The online version contains supplementary material available at 10.1007/s13304-025-02510-9.

## Introduction

The prevention of permanent hypoparathyroidism after total thyroidectomy still remains a major challenge for the endocrine surgeon. This complication impairs patient quality of life while placing a significant burden on healthcare system [1, 2]. Moreover, the true prevalence of long-term hypoparathyroidism, repeatedly underestimated in the literature, has been elucidated through multicenter studies and national registries, reaching figures of up to 12% of thyroidectomies [3].

The prevention of permanent hypoparathyroidism begins well in advance, at the time of the thyroidectomy, through meticulous surgical technique aimed at identifying and preserving all the parathyroid glands in situ and their vascular supply [4–7].

Fewer number of parathyroid glands remaining in situ (PGRIS = 4-(glands in the specimen + autotransplanted glands)) has been shown to have worse short and long-term parathyroid function outcomes by several authors [8–11]. There is broad consensus that parathyroid autotransplantation (PAT) during total thyroidectomy is associated with higher rates of postoperative hypoparathyroidism [9, 12, 13]. The main controversy lies in whether PAT effectively prevents permanent hypoparathyroidism. Some authors suggest that this approach helps prevent long-term hypoparathyroidism at the cost of an increased risk of transient postoperative hypoparathyroidism [14–18], whereas other studies have shown that PAT, not only fails to prevent it but may be actually a risk factor for its development [8, 19–25].

Setting this issue aside, PAT is the accepted approach when in situ preservation is not feasible or when a parathyroid gland is identified within the resected specimen [26]. Among surgeons, there is considerable variation in the approach to PAT, including differences in the indications (from routine use to near avoidance) as well as in the number of glands autotransplanted and the technical aspects of the procedure [27–33].

Our group conducted a survey 10 years ago [33] revealing that indications and surgical technique of PAT varied widely among members of the European Society of Endocrine Surgeons (ESES). Notably, there were considerable differences in timing, preservation methods, fragmentation approaches, implantation sites, and closure methods.

The aim of this questionnaire-based study is to evaluate the current attitudes and practices regarding PAT during thyroid surgery, assessing the impact of indocyanine green (ICG) as indication for PAT and comparing the results with a decade-old survey on the same subject with the same target population.

## Methods

This cross-sectional study evaluated the PAT practices of members of the ESES. The inclusion criteria required active membership in ESES (active, corresponding and junior membership). Honorary members were not included. Using a mailing list provided by the ESES, a structured questionnaire was distributed electronically to eligible participants using Google Forms® platform in October 2024. While the survey was initially administered by the end of 2024, the completion of data analysis occurred in early 2025. Accordingly, to maintain consistency throughout the manuscript, the survey will be designated as the 2025 survey. The results obtained from this survey were compared to those obtained in a previous survey conducted by our group in 2015 among ESES members.

### Questionnaire

The survey consisted of ten closed-ended questions with single or multiple-choice options, addressing PAT indications, frequencies, and intraoperative technical considerations (Table 1). An almost identical questionnaire was previously utilized in a study conducted by our team in 2015 [33] using Survey Monkey® platform. The recent survey introduced two primary modifications: the inclusion of an additional question assessing the proportion of cases in which PAT was utilized, and the addition of indocyanine green as a selectable option for indicating the use of PAT in question 1.

**Table 1:** Responses of the participants to online survey 2025

Question	Choices^1^	n (%)
What are your indications to autotransplant a parathyroid gland? (more than one answer allowed)	Complete devascularization	132 (90.4)
	Recovered from the specimen	105 (71.9)
	Score 0 in ICG (Indocyanine Green)	16 (11.0)
	Change in color	26 (17.8)
	Systematically	1 (0.7)
	Difficult anatomic disposition	36 (24.7)
How often do you perform autotransplantation of parathyroid glands?	Never/Almost never (in less than 1%)	43 (29.4)
	Around 10%	83 (56.8)
	Between 20 and 30%	17 (11.6)
	Between 60 and 80%	3 (2.2)
When do you usually perform the autotransplantation?	Immediately	27 (18.5)
	After completing the lobectomy (within 20 min)	37 (25.3)
	At the end of the procedure (more than 20 min)	82 (56.2)
Where do you keep the gland while waiting for the autotransplantation?	In saline	98 (67.1)
	On a moisted gauze	27 (18.5)
	On a frozen surface	1 (0.7)
	Ice-cold saline	3 (2.0)
	Hartmann’s or balanced salt solution	1 (0.7)
	Nowhere, I do not wait!	15 (10.3)
	Keep in place	1 (0.7)
How do you fragment the gland to be autotransplanted?	I chop it into fragments	97 (66.4)
	I slice it	28 (19.2)
	I triturate it until no fragments are visible	18 (12.3)
	I do not chop it at all	3 (2.1)
What is the average size of the fragments?	Less than 1 mm	70 (47.9)
	From 1 to 3 mm	53 (36.3)
	More than 3 mm	3 (2.1)
	No identifiable fragments	19 (13)
	I do not fragment the gland	1 (0.7)
How do you place the autotransplant tissue into the recipient site?	1 pocket	103 (70.5)
	2 pockets	13 (8.9)
	3 pockets	11 (7.5)
	More than 5 pockets	5 (3.5)
	Injected, no pockets	14 (9.6)
How many fragments do you place per pocket?	1	24 (16.4)
	2	6 (4.1)
	3	13 (8.9)
	4	8 (5.5)
	more than 4	78 (53.4)
	No fragments, I inject	17 (11.7)
Where do you place the autotransplanted tissue?	In the sternocleidomastoid muscle	132 (90.4)
	Strap muscles	11 (7.5)
	Forearm subcutaneous fat	2 (1.4)
	Leaving orthotopically	1 (0.7)
How do you close your pockets?	Non-resorbable stiches	60 (41.1)
	Resorbable stiches	42 (28.8)
	Metallic clips	20 (13.7)
	I do not close them	10 (6.8)
	I do not have pockets, I inject	11 (7.5)
	I seal the small pocket with Ligasure™ or bipolar	3 (2.1)

1. Unless otherwise specified, only single choice was allowed

### Statistical analysis

Categorical variables were summarized as frequencies and percentages. Comparison of proportions was investigated with Chi-square test or Fisher exact test as appropriate. Statistical significance was set at *P* < 0.05.

Responses from surgeons who completed both surveys in 2015 were analyzed, and the concordance of their answers across the two surveys was assessed using Cohen’s Kappa coefficient [34].

## Results

### Survey respondent demographics

**Fig. 1:**
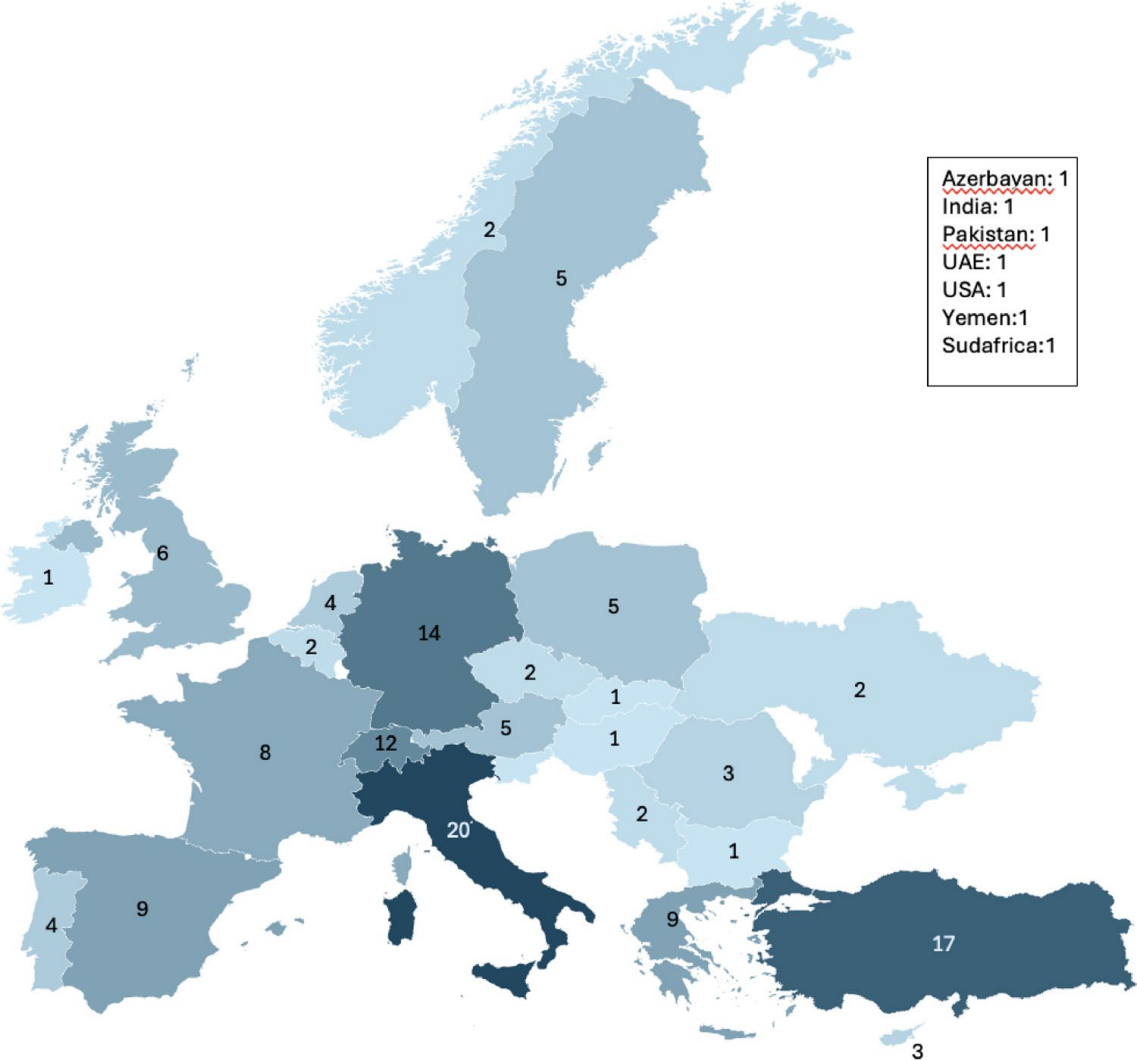
Respondent ESES Surgeons by country

From a total of 490 active and corresponding ESES members (as of November 2024), 146 participants (29.8%) representing 132 centers from 30 countries answered the questionnaire (Fig. 1).

Almost a fourth (22.6%) of the responders were female. Most surgeons practiced in University Hospitals (66.4%) or Public Hospitals (23.3%), which handled a high volume of endocrine surgeries. Private practice accounted only for 10.3% of responders. All surgeons declared themselves experienced and interested in the field of Endocrine Surgery.

### Key findings and survey results

The most common indications for PAT were complete devascularization (90.4%), followed by recovery from the surgical specimen (71.9%) and difficult anatomical disposition (24.7%). Regarding the frequency of autotransplantation, more than half of the responders (56.8%) reported performing PAT in approximately 10% of their thyroidectomies, while 29.4% stated that they rarely or almost never performed the procedure (Table 1).

The timing of autotransplantation varied, with most participants (56.1%) preferring to perform the procedure at the end of surgery (after more than 20 min), while 25.3% performed it within 20 min of completing the thyroidectomy. The most common method for preserving the recovered parathyroid gland before autotransplantation was immersion in saline (66.4%). Other reported practices included placing the gland on moistened gauze (18.4%) or not waiting at all (10.2%).

Regarding tissue preparation, most respondents (66.4%) sliced the gland into small fragments before transplantation, with fragment sizes typically being less than 1 mm (47.9%) or between 1 and 3 mm (36.3%). A minority of pooled surgeons chose to use injection technique.

The most frequently used technique for transplantation was placement in a single muscular pocket (70.5%). Among the 17 surgeons who use the injection technique (mechanically fragmenting the parathyroid tissue and subsequently injecting it into a muscle using a needle), the majority injected the triturated parathyroid tissue directly into the muscle, while three injected the tissue into a pocket.

Additionally, the majority (53.4%) inserted more than four fragments per pocket. The sternocleidomastoid muscle was the preferred transplantation site, chosen by 90.4% of participants. Closure techniques for the pockets varied, with 41% using non-resorbable sutures, 28.8% using resorbable sutures, and 13.6% using metallic clips.

### Comparison with the previous survey

When comparing global responses given in each survey (Table 2), the main change over these years is the implementation of ICG as a new indication for PAT, not present as criteria in 2015. There were no significant differences in the responses given in 2015 and 2025.

**Table 2:** Comparison between global responses in 2015 and 2025 surveys

Question	Choices^1^	2025^2^ n = 146	2015^2^ n = 67	P^3^
What are your indications to autotransplant a parathyroid gland? (more than one answer allowed)	Complete devascularization	132 (90.4)	58 (86.6)	0.112
	Recovered from the specimen	105 (71.9)	47 (70.2)	
	Score 0 in ICG (Indocyanine Green)^5^	16 (11.0)	N/A	
	Change in color	26 (17.8)	17 (25.4)	
	Systematically	1 (0.7)	1 (1.5)	
	Difficult anatomic disposition	36 (24.7)	21 (31.3)	
How often do you perform autotransplantation of parathyroid glands?^5^	Never/Almost never (in less than 1%)	43 (29.4)	N/A	
	Around 10%	83 (56.8)		
	Between 20 and 30%	17 (11.6)		
	Between 60 and 80%	3 (2.2)		
When do you usually perform the autotransplantation?	Immediately	27 (18.5)	13 (19.8)	0.912^4^
	After completing the lobectomy (within 20 min)	37 (25.3)	17 (25.8)	
	At the end of the procedure (more than 20 min)	82 (56.2)	36 (54.6)	
			1 NR	
Where do you keep the gland while waiting for the autotransplantation?	In saline	98 (67.1)	46 (68.7)	0.500
	On a moistened gauze	27 (18.5)	11 (16.4)	
	Nowhere, I do not wait!	15 (10.3)	6 (9.0)	
	In ice-cold saline	3 (2.0)	0	
	In Hartmann’s or balanced salt solution	1 (0.7)	0	
	On a frozen surface	1 (0.7)	4 (6.0%)	
	Keep in place	1 (0.7)	0	
How do you fragment the gland to be autotransplanted?	I chop it into fragments	97 (66.4)	37 (56.9)	0.753
	I slice it	28 (19.2)	17 (26.2)	
	I triturate it until no fragments are visible	18 (12.3)	11 (16.9)	
	I do not chop it at all	3 (2.1)	0	
			2 NR	
What is the average size of the fragments?	Less than 1 mm	70 (47.9)	30 (45.5)	0.903
	From 1 to 3 mm	53 (36.3)	29 (43.9)	
	More than 3 mm	3 (2.1)	0	
	No identifiable fragments	19 (13)	7 (10.6)	
	I do not fragment the gland	1 (0.7)	0	
			1 NR	
How do you place the autotransplant tissue into the recipient site?	1 pocket	103 (70.5)	37 (57.8)	0.052
	2 pockets	13 (8.9)	9 (14.1)	
	3 pockets	11 (7.5)	3 (4.7)	
	More than 3 pockets	5 (3.5)	10 (14.9)	
	Injected, no pockets	14 (9.6)	8 (12.5)	
How many fragments do you place per pocket?	1	24 (16.4)	8 (12.5)	0.566
	2	6 (4.1)	5 (8.2)	
	3	13 (8.9)	3 (4.9)	
	4	8 (5.5)	7 (11.5)	
	More than 4	78 (53.4)	28 (45.9)	
	No fragments, I inject	17 (11.7)	10 (16.4)	
			6 NR	
Where do you place the autotransplanted tissue?	Sternocleidomastoid muscle	132 (90.4)	62 (98.4)	0.399
	Strap muscles	11 (7.5)	0	
	Forearm subcutaneous fat	2 (1.4)	0	
	Leave it orthotopically	1 (0.7)	0	
	In the muscles of forearm	0	1 (1.6)	
			4 NR	
How do you close your pockets?	Non-resorbable stiches	60 (41.1)	24 (36.9)	0.830
	Resorbable stiches	42 (28.8)	20 (30.8)	
	Metallic clips	20 (13.7)	8 (12.3)	
	I do not close them	10 (6.8)	5 (7.7)	
	I do not have pockets, I inject	11 (7.5)	8 (12.3)	
	I seal the small pocket with Ligasure™ or bipolar	3 (2.1)	0	
			2 NR	

*NR* non responders, *N/A* option not available/not cited in the survey

1. Unless otherwise specified, only single choice was allowed; 2. N (%) for all columns; 3. Fisher exact test unless otherwise specified 4. Chi-square test. 5. Question/Option not available in the 2015 survey

Regarding the indication for PAT, the number of criterion chosen were similar in both surveys regardless of the period. One criteria for PAT was selected by 27% of responders in 2015 and by 25.3% in 2025, two criteria in 40% and 39%, respectively and, 3 or more criteria were chosen by 33% of responders in 2015 and 35% ten years later. Similar answers were reported in terms of timing of PAT, the site where the parathyroid gland was kept until its autotransplantation and the size of fragments.

Ten years ago, pooled surgeons trend to slice fragments more than now and less chopped into fragments. Injected technique was 1.4 fold less frequent in 2015. The chosen place of PAT still remains the sternocleidomastoid muscle (SCM), reported in 98.4%, and forearm only reported by one surgeon.

Table 3 reflects the responses of the 20 participants who completed both, the 2015 and 2025 surveys. Although the responses did not exhibit significant differences over the 10-year period, none of the surgeons answered exactly the same, indicating that all participants had changed their technical approach (Supplementary material).

**Table 3:** Comparison of answers given by the 20 participants in both surveys (2015 and 2025)

Question	Choice^1^	2015^2^ 20	2025^2^ 20
What are your indications to autotransplant a parathyroid gland? (more than one answer allowed)	Complete devascularization	20 (100)	19 (95.0)
	Recovered from the specimen	14 (70)	15 (75.0)
	Score 0 in ICG (Indocyanine Green)	0^3^	3 (15.0)
	Change in color	3 (15.0)	3 (15.0)
	Difficult anatomic disposition	3 (15.0)	4 (20.0)
When do you usually perform the autotransplant?	Immediately	5 (25.0)	5 (25.0)
	After completing the lobectomy (within 20 min)	6 (30.0)	3 (15.0)
	At the end of the procedure (more than 20 min)	9 (45.0)	12 (60.0)
Where do you keep the gland while waiting for the autotransplant?	In saline	13 (65.0)	13 (65.0)
	On a moistened gauze	5 (25.0)	4 (20.0)
	Nowhere, I do not wait!	2 (10.0)	3 (15.0)
How do you fragment the gland to be autotransplanted?	I chop it into fragments	11 (55.0)	14 (70.0)
	I slice it	6 (30.0)	4 (20.0)
	I triturate it until no fragments are visible	3 (15.0)	2 (10.0)
What is the average size of the fragments?	Less than 1 mm	11 (55.0)	13 (65.0)
	From 1 to 3 mm	7 (35.0)	3 (15.0)
	More than 3 mm	0	1 (5.0)
	No identifiable fragments	2 (10.0)	3 (15.0)
How do you place the autotransplant tissue into the recipient site	1 pocket	12 (60.0)	15 (75.0)
	2 pockets	3 (15.0)	0
	3 pockets	2 (10.0)	2 (10.0)
	Injected, no pockets	3 (15.0)	3 (15.0)
How many fragments do you place per pocket?	1	3 (15.0)	2 (10.0)
	2	2 (10.0)	0
	3	3 (15.0)	2 (10.0)
	4	1 (5.0)	2 (10.0)
	more than 4	8 (40.0)	11 (55.0)
	No fragments, I inject	3 (15.0)	3 (15.0)
Where do you place the autotransplanted tissue?	In the sternocleidomastoid muscle	18 (90.0)	18 (90.0)
	Strap muscles	2 (10.0)	1 (5.0)
	Forearm subcutaneous fat	0	1 (5.0)
How do you close your pockets?	Non-resorbable stiches	9 (45.0)	8 (40.0)
	Resorbable stiches	5 (25.0)	7 (35.0)
	Metallic clips	3 (15.0)	2 (10.0)
	I do not close them	0	2 (10.0)
	I do not have pockets, I inject	3 (15.0)	1 (5.0)

1. Unless otherwise specified, only single choice was allowed; 2. N(%) for all columns; 3. Option not available in the 2015 survey

**Fig. 2:**
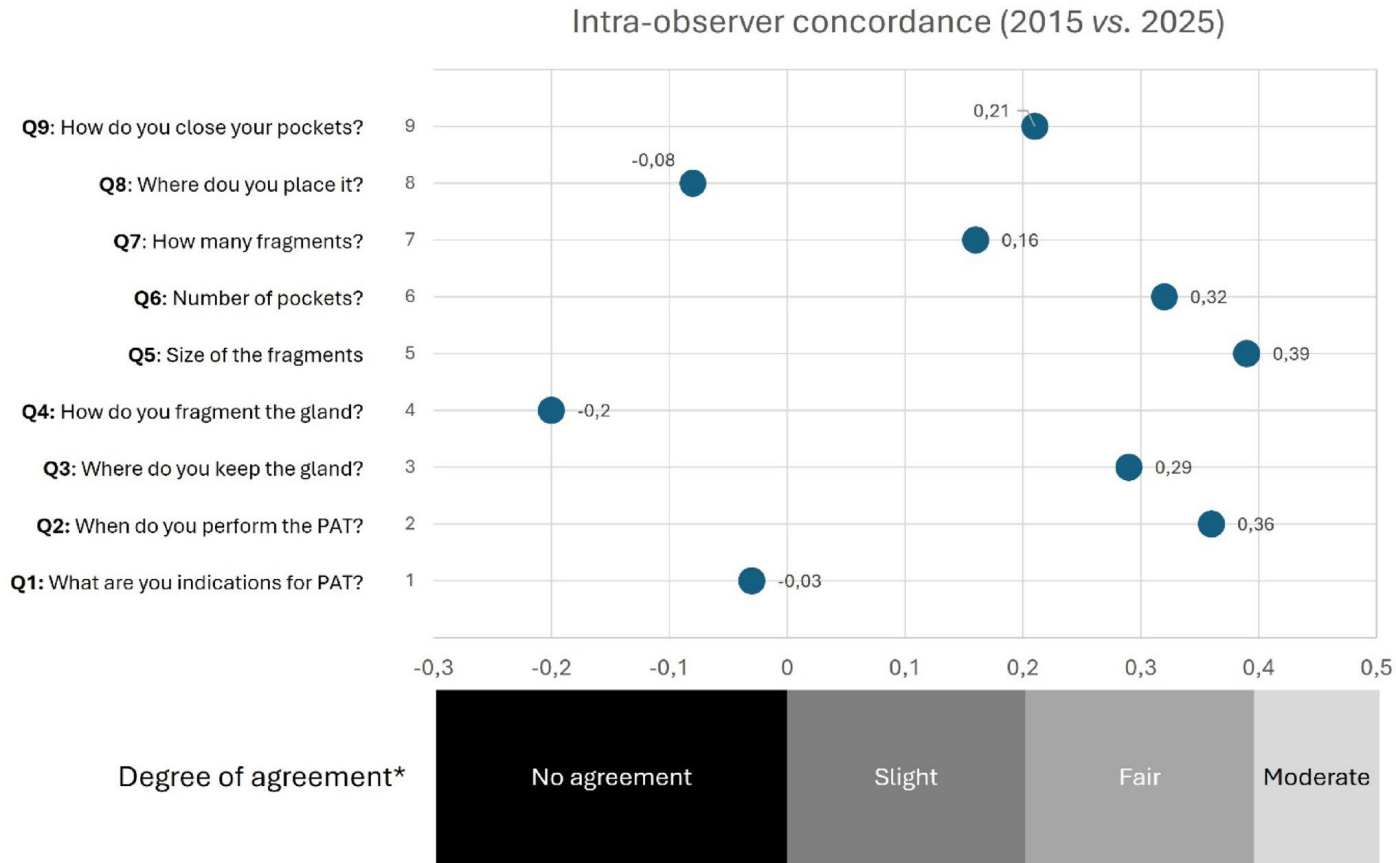
Concordance of the answers given by surgeons who responded both surveys, in 2015 and 2025, assessed using Cohen’s Kappa coefficient

The majority of questions showed a slight to moderate agreement among responses given by the 20 surgeons in 2015 and 2025 (Fig. 2). The Cohen’s Kappa index showed no agreement in questions 1 (*What are your indications to autotransplant a parathryoid gland?*), 4 (*How do you fragment the gland to be autotransplanted?*) and 8 (*Where do you place the autotransplanted tissue?*).

In Q1, the agreement of responses given in 2015 and 2025 was 15% with a Kappa -0.03 (concordance worse than chance), probably influenced by the multiple-choice option and disparity of responses. Similar concordance was found in Q4, with an observed concordance of 35%. In Q8, 80% of surgeons responded “SCM” in both years, which results in a high observed concordance but negative Kappa value.

## Discussion

This is the first study evaluating the indications and technique of PAT during thyroidectomy performed by the Endocrine Society European Surgeons. In line with the absence of widely accepted guidelines for PAT indications and techniques, we observed significant variation among ESES surgeons in various aspects of PAT.

### Indications and frequency of parathyroid autotransplantation

More than 86% of the responders reported they performed PAT in 10% or less of their thyroidectomies whereas only 2% did so in 60–80% of their cases. Only one responder reported performing PAT systematically.

The trailblazers of PAT were Lahey in 1926, commonly cited as the pioneer of PAT during thyroidectomy in humans [35] and Alveryd [36] who in the late 1960s performed careful anatomical studies of the parathyroid glands and did advocate PAT using the whole gland intramuscular implantation technique. In his initial report, however, 7/32 patients undergoing PAT (one or more glands) developed permanent hypoparathyroidism. Four-gland autotransplantation afforded some benefit compared to fewer transplanted glands.

Since Wells’ group published a seminal paper in 1976 [37] showing that small fragments of hyperplastic parathyroid tissue transplanted into muscle pockets would function and prevent hypoparathyroidism in MEN 1 patients after total parathyroidectomy, the concept was extrapolated to autotransplanted normal parathyroid tissue assuming it could survive and secrete parathormone and prevent permanent hypoparathyroidism after total thyroidectomy. Therefore, in the 1990s-2000s some groups advocated for liberal and even routine use of PAT [15, 32, 38, 39], claiming the routine autotransplantation of at least one removed parathyroid gland can decrease the incidence of permanent hypoparathyroidism to less than 1% [17, 32].

Years later, the same authors published data suggesting that they were no longer in favour of such radical approach [40, 41]. The Sydney group reported the need for chronic calcium replacement in a cohort of patients after total thyroidectomy and liberal parathyroid autografting (mean of 1.4 transplanted glands per patient) [41].

With the years, attitude regarding autotransplantation has trended towards more conservative, in line with our data. The systematically indication of PAT was twofold 10 years ago when comparing results from 2015 and 2025 surveys.

In search of other solutions, cryopreservation has been proposed to avoid permanent hypoparathyroidism following parathyroid or thyroid surgery. Cryopreservation involves the freezing and storage of parathyroid tissue in case permanent hypoparathyroidism occurs. Autotransplantation of cryopreserved tissue is less effective than immediate autotransplantation with a highly variable reported success rate from 10–100% [42–44]. The benefit of retaining parathyroid tissue for cryopreservation following the majority of cases of thyroidectomy remains doubtful [45].

Complete devascularization and recovery from the surgical specimen were the most commonly reported indications for PAT. The indication of PAT when the parathyroid has been accidentally excised is undoubtably accepted by the majority of authors and guidelines [26], however it was reported as indication by 72%, instead of an expected 100% by polled surgeons.

The threshold for PAT in cases of parathyroid glands with difficult anatomic disposition, chosen as an indication by 25% of responders, it is sometimes subjective and may be influenced by the belief of the surgeon in the viability and functional capacity of the grafted tissue. Cui et al. [46], in a thorough study assessing the anatomy of parathyroid vascularization, showed that in approximately one-quarter of cases, thyroidectomy may lead to partial or complete devascularization of the parathyroid glands and suggested that in situ preservation of such glands may not effectively prevent permanent hypoparathyroidism, making PAT a viable alternative in these cases. Our group found that permanent hypoparathyroidism in patients undergoing PAT was threefold higher than that observed in patients not autografted (9.8 *vs.* 3.1%; *P* < 0.002) [8]. In a subsequent study analyzing 186 patients with three parathyroid glands in situ (PGRIS 3) after total thyroidectomy, sample was divided in two groups: one in which the fourth gland had been autotransplanted into the ipsilateral sternocleidomastoid muscle and another in which the fourth gland had been inadvertently resected. The rates of immediate, protracted (1-month) and permanent parathyroid failure were the same regardless of whether the fourth gland was autotransplanted or inadvertently sent to the Pathology lab [23]. It should be noted that in these studies, the vascularization status of the remaining in situ parathyroid glands was not assessed, representing a significant limitation.

Some studies other than our group have found PAT to be a predicting factor for permanent hypoparathyoridism.

A recent meta-analysis [19] showed that, when assessing 12 studies that defined as hypoparathyroidism beyond 12 months, patients with PAT had 71% more risk of having permanent hypoparathyroidism (OR 1.71 (1.49–1.96)). There was, however, high heterogeneity in data presentation, hypoparathyroidism definition, and inconsistencies between PAT and permanent hypoparathyroidism among the studies, so the authors conclude the results should be interpreted with caution.

In a retrospective multicenter cohort study performed in 10 hospitals in the Southwestern region of the Netherlands, assessing 749 patients, Lončar et al. Showed that patients with AT had 2.85.

times more risk to develop permanent hypoparathyroidism OR 2.85 (1.057–7.687) [20].

Another study coming from Sweeden assessed 938 patients from the Scandinavian Quality Register for Thyroid, Parathyroid and Adrenal Surgery and the Swedish National Patient Register. Higher risk of permanent hypoparathyroidism was found in patients with parathyroid autotransplantation (OR 1.72 (1.47–2.01)) [24].

Promberger et al. [25] also published an increase risk of permanent hypoparathyroidism with an OR of 2.018 (1.021–3.985) when one or two glands were autotransplanted.

According to these results, our attitude towards PAT is really restricted and all efforts are focused on leaving parathyroid glands in situ, reserving PAT only for accidentally excised glands.

Nearly one-fifth of respondents identified parathyroid gland discoloration as the third most frequent indication for PAT. PAT of discolored glands (or of the most discolored one) has been widely adopted in the regular practice. A study by Promberger et al. [47] demonstrated that dysfunction of discolored parathyroid glands is typically transient, with sequential recovery occurring within a short period postoperatively. They advised against using discoloration alone as an indication for PAT. Furthermore, change in color may not be a reliable marker of parathyroid dysfunction. Similarly, Lang et al. [48] reported a higher rate of permanent hypoparathyroidism in cases in which no color change was detected in any of the four parathyroid glands than in cases where discoloration of one or two glands was noticed. An alternative method for assessing parathyroid gland viability is the fine-needle pricking test which has been suggested as an adjunctive tool in determining the need for PAT [14, 21]. Fine-needle pricking consists on gently puncturing the surface of the parathyroid gland with a fine needle to observe its vascular response. When the gland is viable and adequately vascularized, a small droplet of blood appears promptly at the site of the puncture, indicating intact perfusion. Reduced or absence of vascular supply determined by green indocyanine fluorescence angiography was proposed as an indication for PAT following the publication of Vidal et al. in 2016 [49].

As a result, an increase of rate of PAT has been observed during the last 10 years. Karahan et al*.* [50] revealed that a significantly higher proportion of patients in the ICG group underwent parathyroid gland autotransplantation compared to the conventional group (10% *vs.* 1.7%, *p* < 0.01). Likewise, Rudin et al*.* [51] compared 86 patients with ICG to 120 without ICG and the parathyroid autotransplantation rate. Significantly higher in the ICG group (36% *vs.* 12%).

Only 11% of the responders reported ICG score 0 as an indication for PAT. Unfortunately, the number of polled surgeons who use ICG in their regular practice was unknown since we did not gather these data and therefore, we do not know the proportion of indication of PAT in that group.

### Technical details of PAT

Although implantation of fragmented parathyroid tissue into pockets within the ipsilateral sternocleidomastoid muscle has been the predominant technique for decades, no standardized protocol exists for the technical aspects of PAT [22, 33].

The classical PAT technique described by Wells’ group [15], during thyroidectomy for nonfamilial thyroid disease, reported that every attempt was made to reserve at least one parathyroid gland. However, if there was any question regarding parathyroid viability or if a parathyroid gland was identified within the resected specimen, it was immediately placed in iced saline until the end of the operation (< 120 min). One or two parathyroid glands were sliced into 1 × 3 mm pieces and three to four slices of normal parathyroid were implanted into separate muscle pockets in the sternocleidomastoid muscle. A total of 20 pieces were grafted distributed into 5 pockets. Each muscle pocket was closed with a black silk suture.

As can be observed in our results, PAT technique has evolved and surgeons employ various other methods for graft preparation, including preservation, fragmentation through mincing, chopping or direct implantation or injection of parathyroid suspensions into different intramuscular or subcutaneous pockets in varying numbers. Surprisingly, the majority (70.5%) reflected only one pocket was used to put the parathyroid tissue and only 11% placed the autotransplanted tissue into 3 pockets or more.

More than half of the responders, waited until the end of the surgery (more than 20 min) to perform the PAT. There is no study assessing the impact of timing in parathyroid tissue viability although some experts recommend tissue should ideally be reimplanted as early after sacrifice as possible and every effort should be made to avoid the diseccation that can occur under the intense theater illumination if not immediately placed in a balanced salt solution [52].

A minority of pooled surgeons chose to use an injection technique. Gauger et al. [53] explored an alternative PAT technique comparing the injection of dispersed parathyroid tissue into the ipsilateral sternocleidomastoid muscle with the standard fragmented technique. They found no differences in terms of long-term iPTH concentrations.

The preferred site for PA during thyroid surgery was the SCM, as showed by a systematic review performed by Iorio et al. [31], since it can be reached by the same approach used for thyroidectomy, it is highly vascularized and can provide an easy approach for the pocket made to receive the fragmented glands.

### Comparison between 2015 and 2025 surveys

When comparing global responses, the main change along these years is the implementation of ICG score to guide the decision to autotransplant a parathyroid gland.

As previously mentioned, 20 participants who responded to the surveys in 2015 and 2025 reflected some changes in their technical approaches over the 10-year period.

The Cohen’s Kappa index showed no agreement in questions 1 (*What are your indications to autotransplant a parathryoid gland?*), 4 (*How do you fragment the gland to be autotransplanted?*) and 8 (*Where do you place the autotransplanted tissue?*). In Q1, the agreement of responses given in 2015 and 2025 was 15% with a Kappa − 0.03 (concordance worse than chance), probably influenced by the multiple-choice option and disparity of responses. Similar concordance was found in Q4, with an observed concordance of 35% and Kappa concordance worse than expected by chance. Lastly, in Q8, the majority (80%) responded “SCM” in both years, which results in a high observed concordance. However, this also leads to a high expected concordance by chance, which, in turn, reduces the Kappa value. Kappa value can be negative or very low when there is little variability in the responses, one or two categories dominate the data, or when there are only minor changes outside the dominant category. Thus, even if there appears to be substantial agreement visually, the Kappa index penalizes this due to the high probability of agreement occurring by chance.

### Role of parathyroid autotransplantation and lack of standardization

Given these diverse technical approaches, it remains challenging to define a universally accepted standard for parathyroid preservation, preparation, and reimplantation. Furthermore, the impact of each technique on PAT success remains uncertain due to the limited number of comparative studies. Although both short-term and long-term PAT outcomes have been extensively studied [30], the influence of technical variations has not been thoroughly investigated.

### Limitations

This study has several limitations. First, the survey-based design inherently introduces response bias, as participation was voluntary and responses may not fully reflect actual surgical practices. Second, the study was limited to the members of ESES, potentially restricting generalizability to endocrine surgeons in other regions with different practice patterns. Third, the questionnaire did not assess long-term functional outcomes of PAT, such as graft viability and postoperative parathyroid function, which are crucial for evaluating its clinical effectiveness. Additionally, the study did not explore the potential impact of surgeon experience, institutional protocols, or adjunctive intraoperative techniques on PAT outcomes. The level of evidence in this study is relatively low, as the findings are primarily based on expert preferences rather than empirical data. Another issue is the subjectivity in determining indications for PAT, such as using color changes or ICG fluorescence as criteria. Studies have questioned the reliability of these indicators, as some discoloration might not correlate with dysfunction [47]. The study also introduce ICG as additional option to indicate the autotransplantation, however, the absence of this option in previous survey and small number of responders choosing this option does not allow to get deeper into its effectiveness, leaving room for further research.

Additionally, while the survey provides valuable descriptive statistics, it lacks prospective clinical outcomes, which would have strengthened the discussion on whether varying PAT techniques lead to differences in long-term patient health.

Future prospective, multicenter studies incorporating objective outcome measures assessing long-term functional outcomes of PAT are needed to better understand the optimal indications and techniques for PAT.

## Conclusions

This study highlights the substantial variability in indications and details of surgical technique for PAT among ESES surgeons, reflecting the absence of standardized guidelines. While complete devascularization and recovery from the surgical specimen were the most common indications, approaches to preservation, fragmentation, implantation, and closure techniques varied widely. These discrepancies may contribute to the ongoing debate regarding PAT’s effectiveness in preventing postoperative hypoparathyroidism. Given the lack of consensus on the optimal technique and the limited data on long-term outcomes, future studies should focus on evaluating the impact of different surgical approaches on graft survival and patient outcomes to establish standardized best practices.

## Supplementary Information

Below is the link to the electronic supplementary material.

Supplementary file1 (DOCX 915 kb)
