# Supplementary material for: Parathyroid autotransplantation during thyroidectomy among the European Society of Endocrine Surgeons: we still do not agree
Source: Updates Surg. 2026 Mar 17;78(3):1269–80. doi: 10.1007/s13304-025-02510-9 (PMC13249698; doi:10.1007/s13304-025-02510-9)
Supplement: Supplementary file 1 — Supplementary file1 (DOCX 915 kb) [file 13304_2025_2510_MOESM1_ESM.docx]

**Supplementary Material**

Observed concordance of the answers responded by the 20 surgeons who participate in both surveys, 2015 and 2025

Concordance of answers to Q1 (2015 *vs.* 2025)

What are your indications to autotransplant a parathyroid gland?


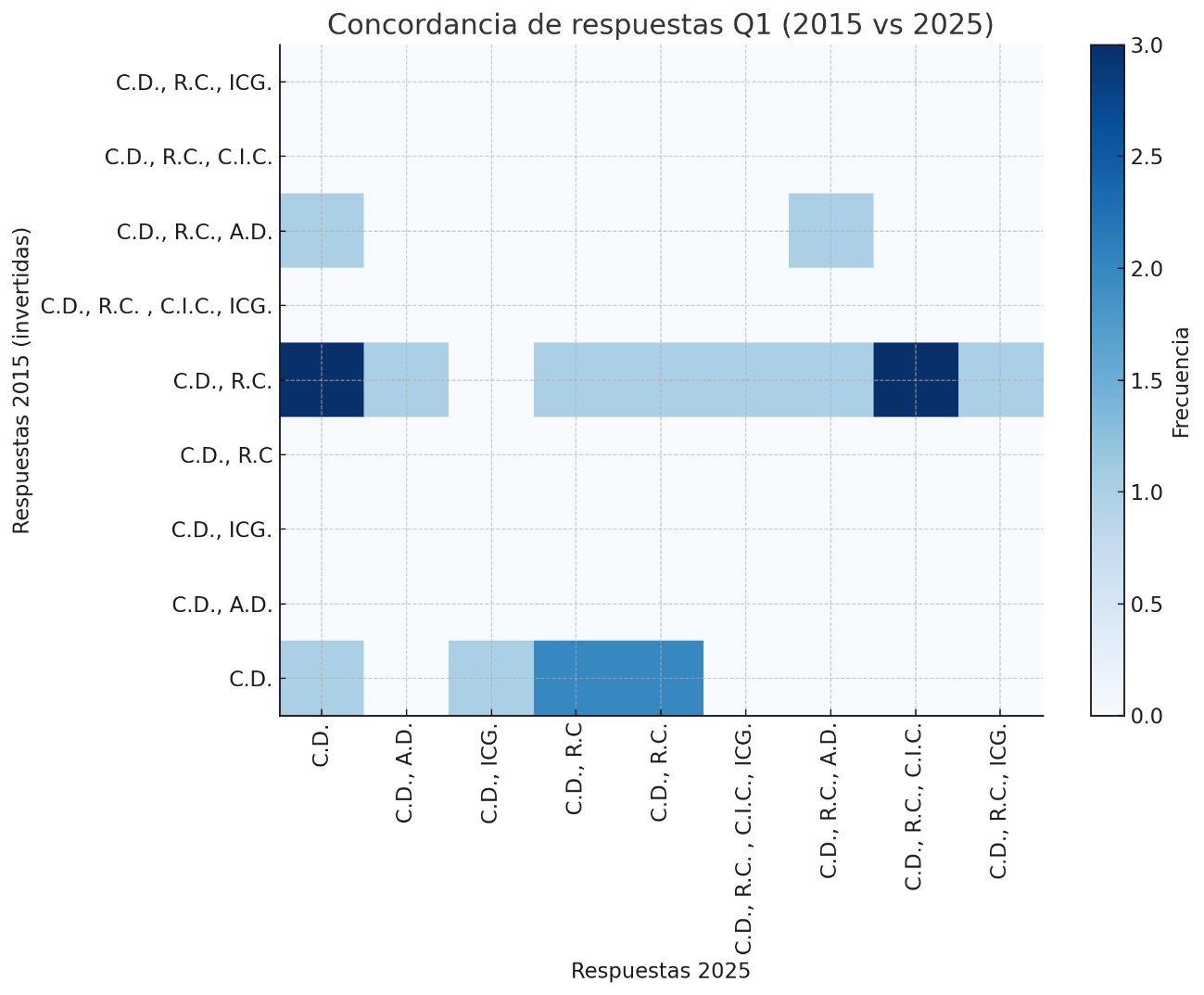


Answers 2015

Frequency

Answers 2025

C.D.: complete devascularization, R.C.: recovered from the specimen, C.I.C.: change in color, ICG.: score 0 in indocyanine green, A.D.: anatomic disposition.

Concordance of answers to Q2 (2015 *vs.* 2025)

When do you usually perform the autotransplant?


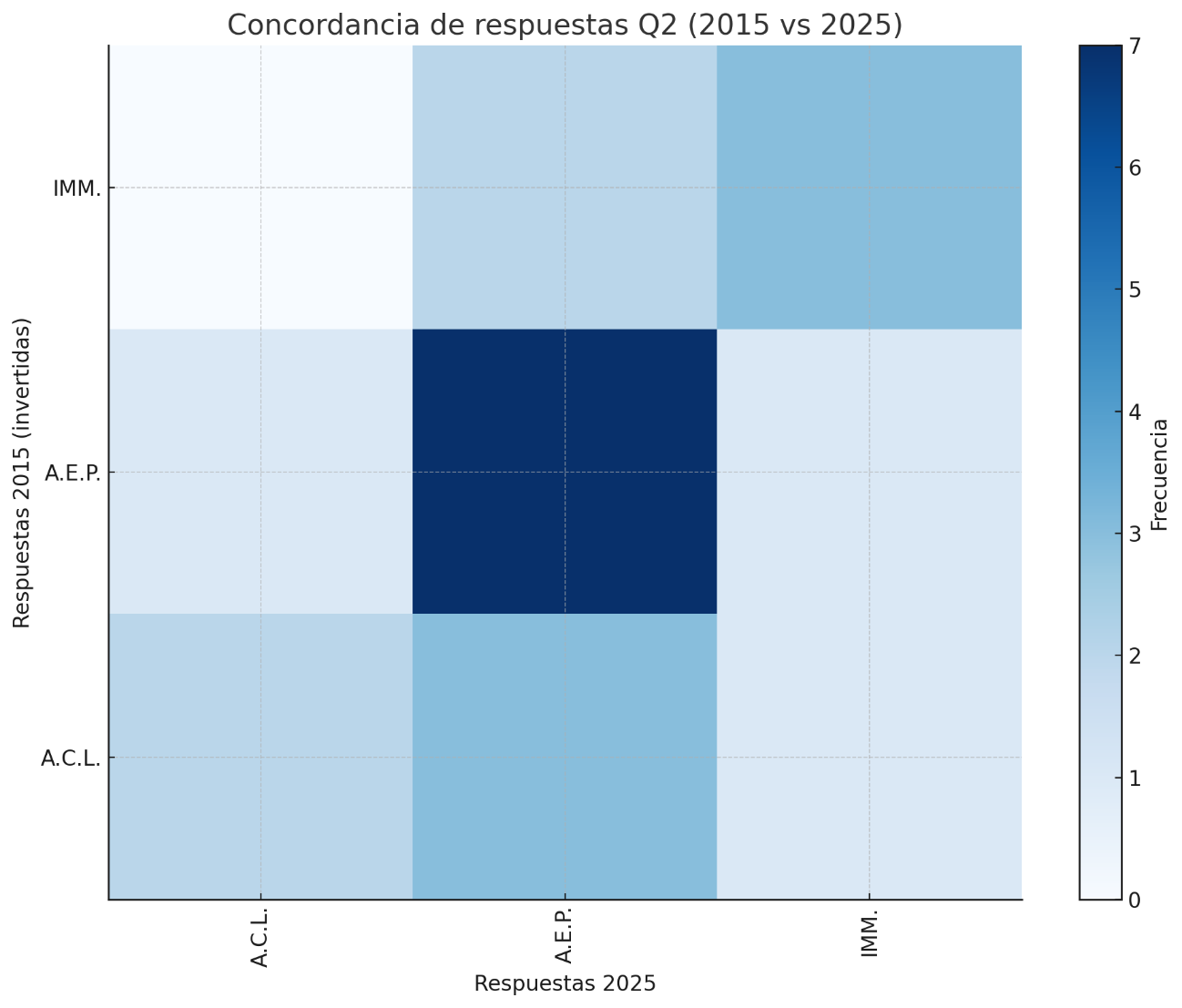


Answers 2015

Answers 2025

Frequency

A.E.P.: at the end of the procedure (more than 20 minutes), IMM.: immediately, A.C.L.: after completing the lobectomy (within 20 minutes)

Concordance of answers to Q3 (2015 *vs.* 2025)

Where do you keep the gland while waiting for the autotransplant?


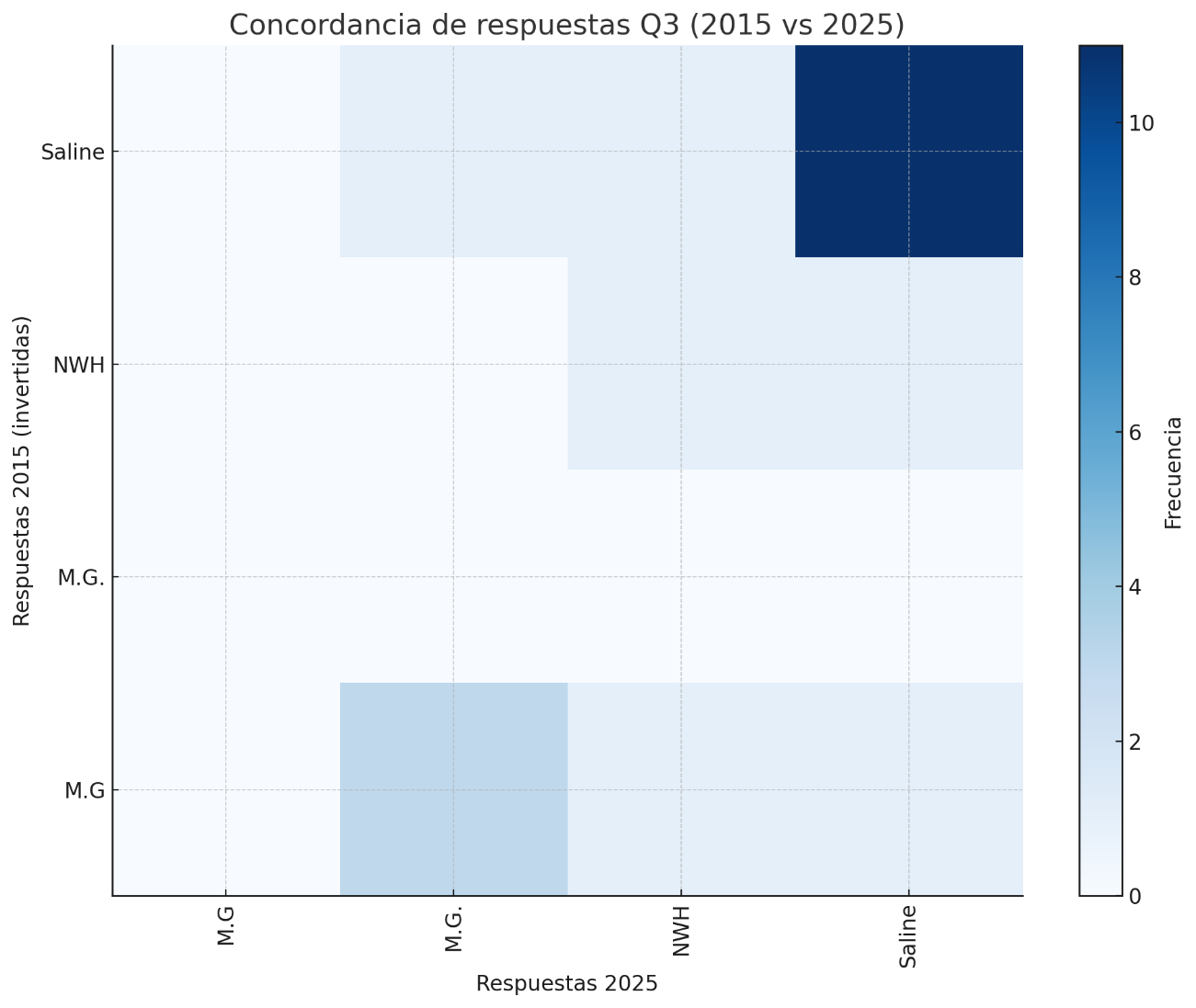


Answers 2025

Answers 2015

Frequency

NWH: nowhere, I do not wait, M.G.: on a moistened gauze

Concordance of answers to Q4 (2015 *vs.* 2025)

How do you fragment the gland to be autotransplanted?


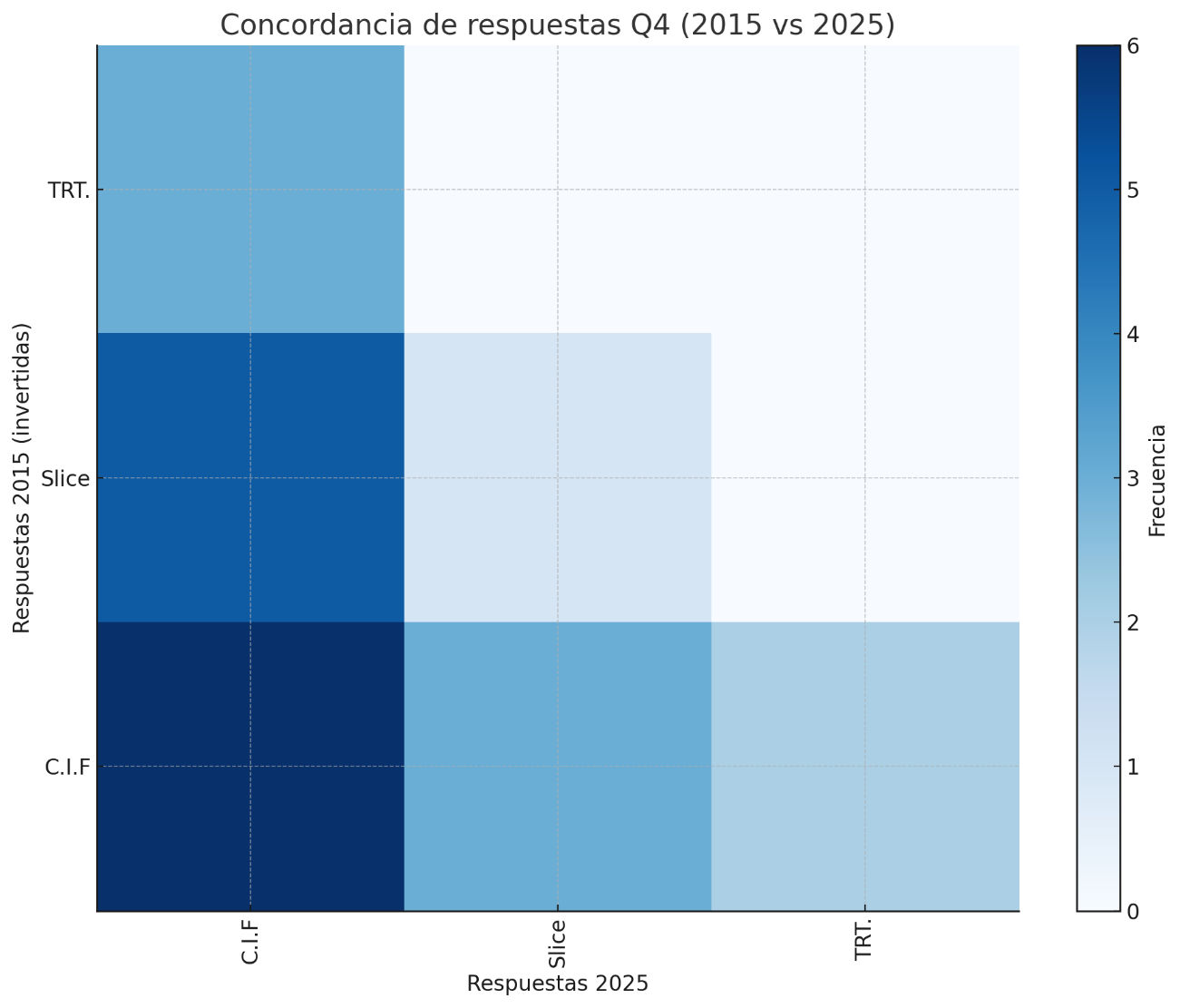


Answers 2025

Answers 2015

Frequency

C.I.F: I chop it into fragments, TRT.: I triturate it until no fragments are visible

Concordance of answers to Q5 (2015 *vs.* 2025)
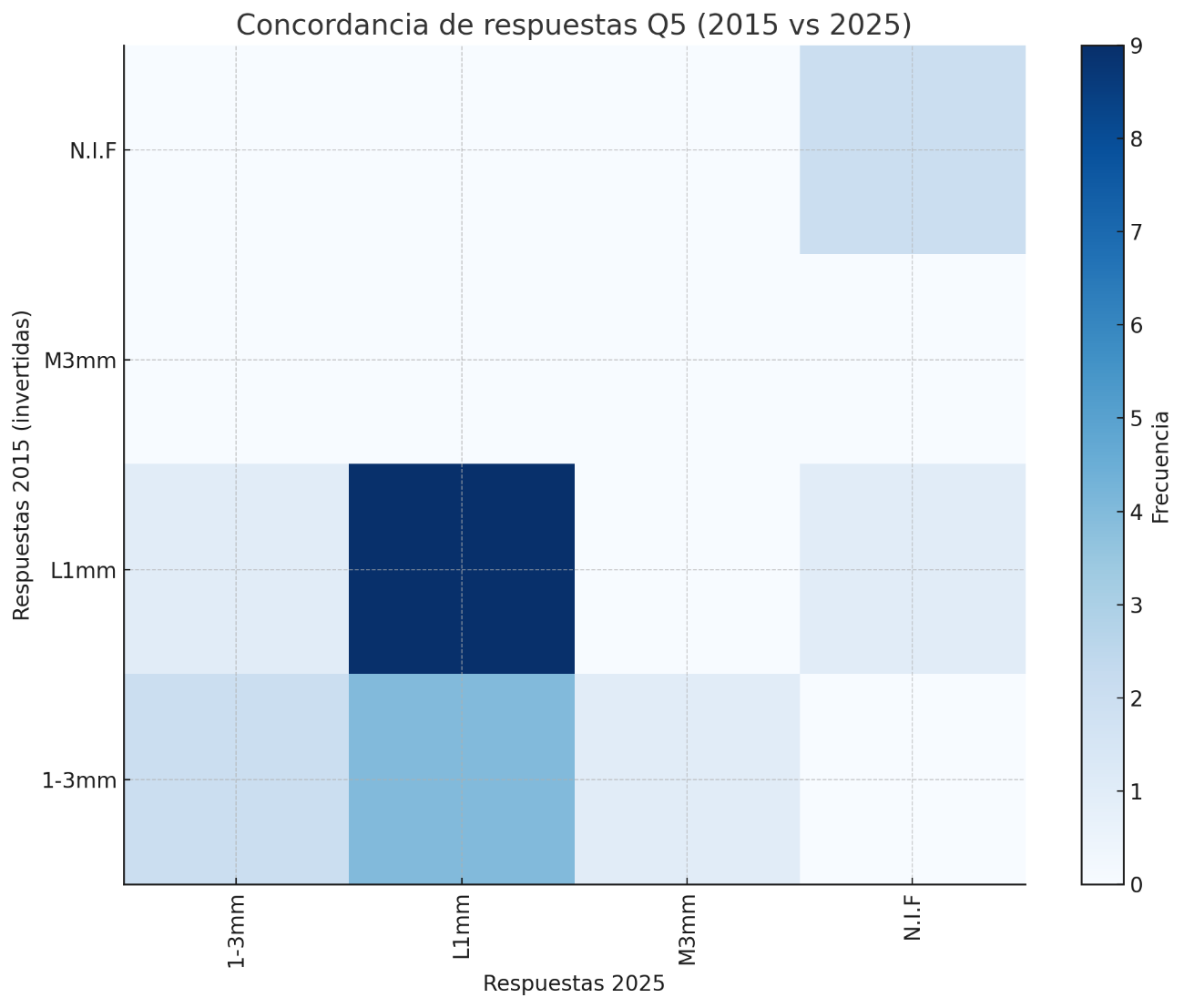


Frequency

Answers 2025

Answers 2015

What is the average size of the fragments?

L1mm: less than 1 mm, M3mm: more than 3 mm, N.I.F: no identifiable fragments

Concordance of answers to Q6 (2015 *vs.* 2025)

How do you place the autotransplant tissue into the recipient site?


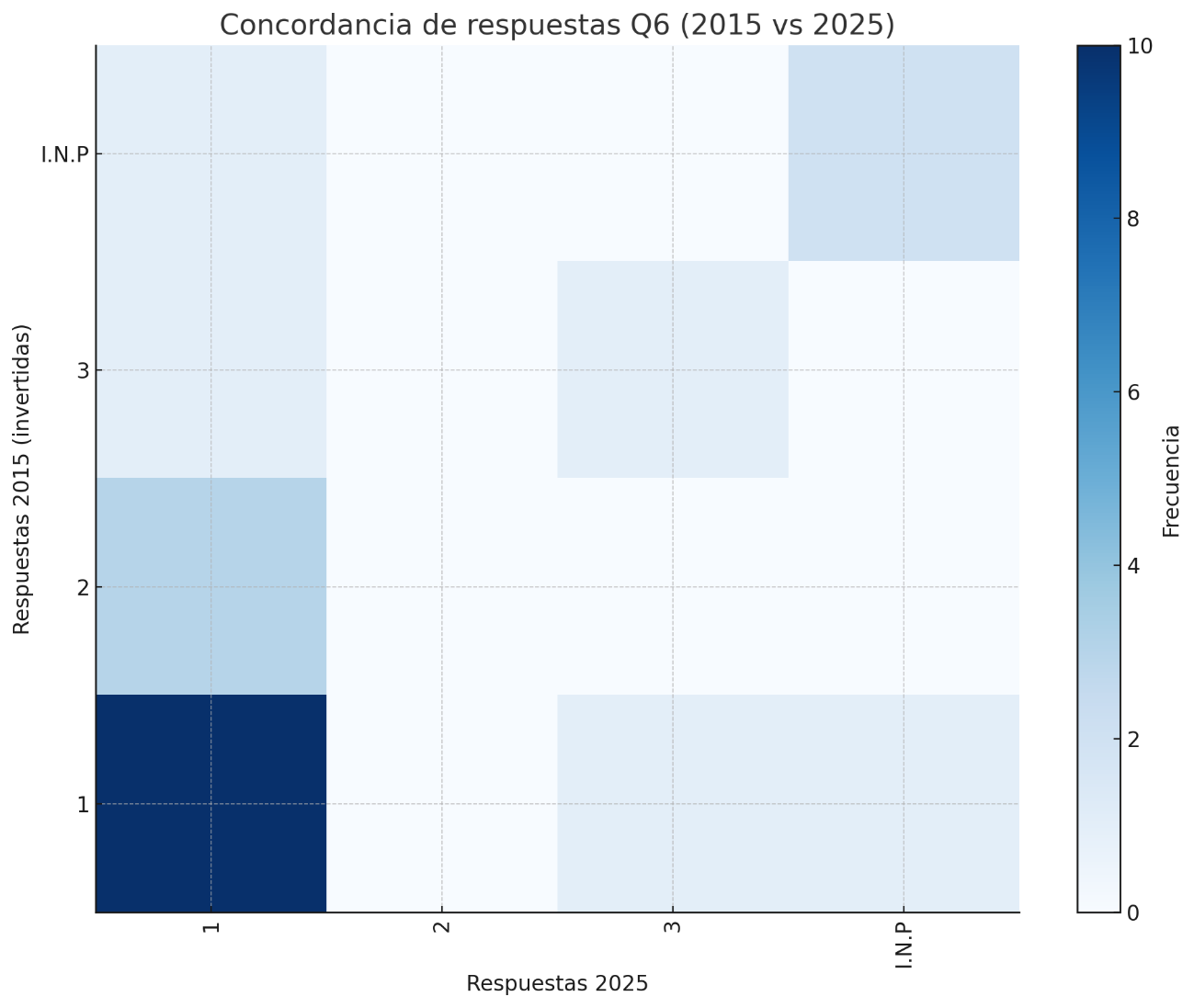


Answers 2025

Frequency

Answers 2015

Number of pockets; I.N.P: injected, no pockets

Concordance of answers to Q7 (2015 *vs.* 2025)

How many fragments do you place per pocket?


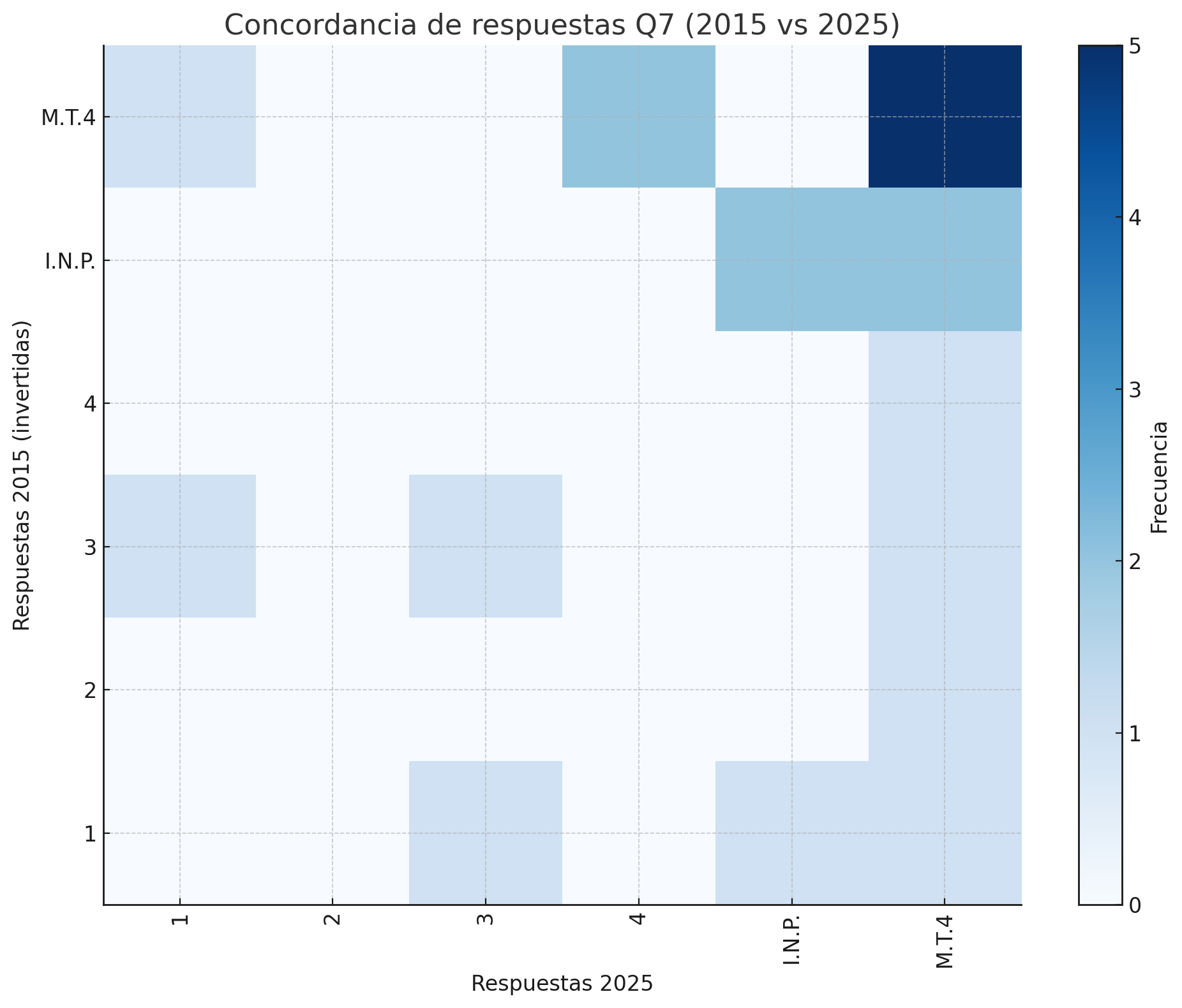


Answers 2025

Frequency

Answers 2015

Number of fragments; M.T.4: more than 4, I.N.P.: no fragments, I inject.

Concordance of answers to Q8 (2015 *vs.* 2025)

Where do you place the autotransplanted tissue?


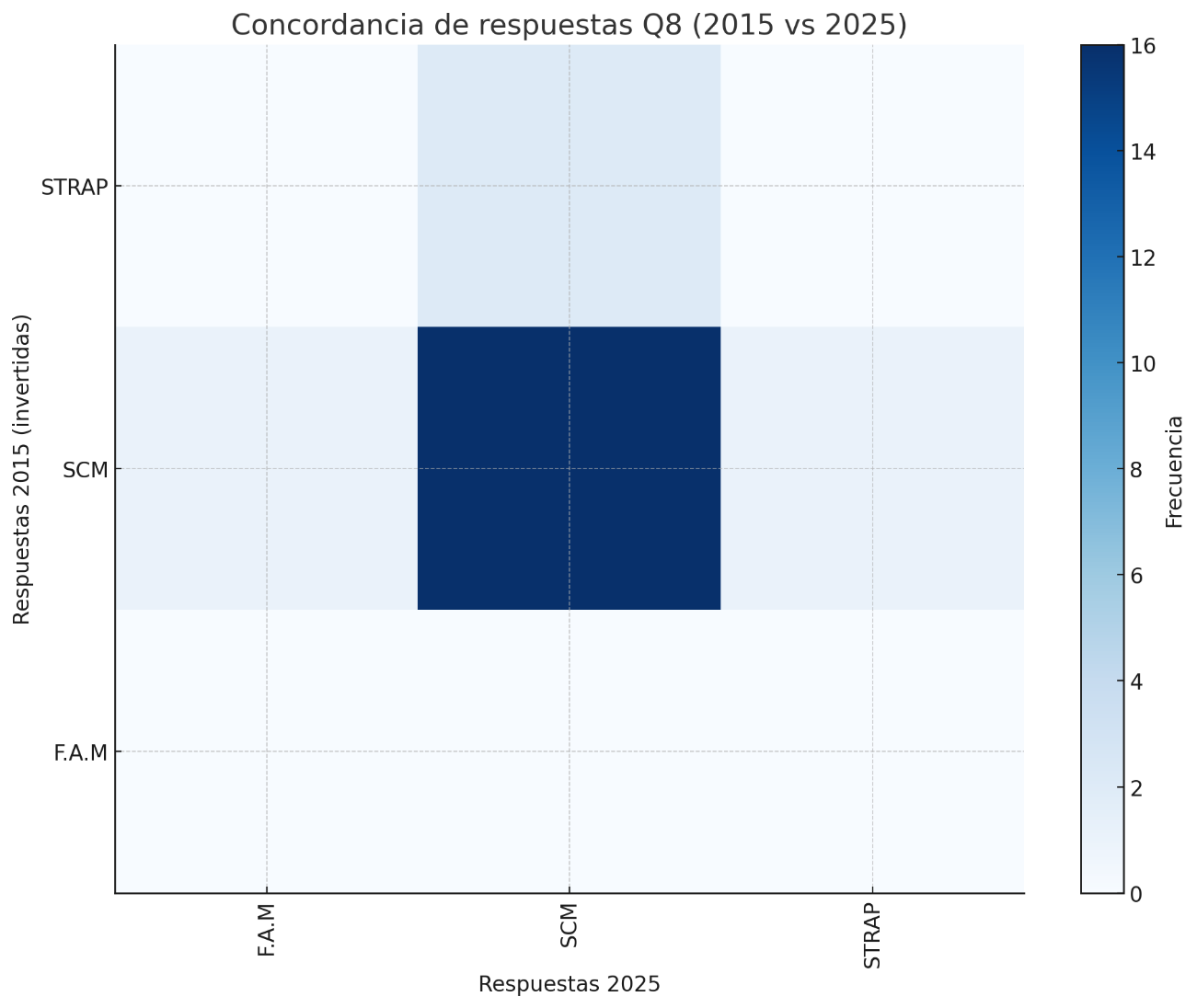


Answers 2025

Answers 2015

Frequency

SCM: sternocleidomastoid muscle, F.A.M: forearm subcutaneous fat, STRAP: strap muscles

Concordance of answers to Q9 (2015 *vs.* 2025)

How do you close your pockets?


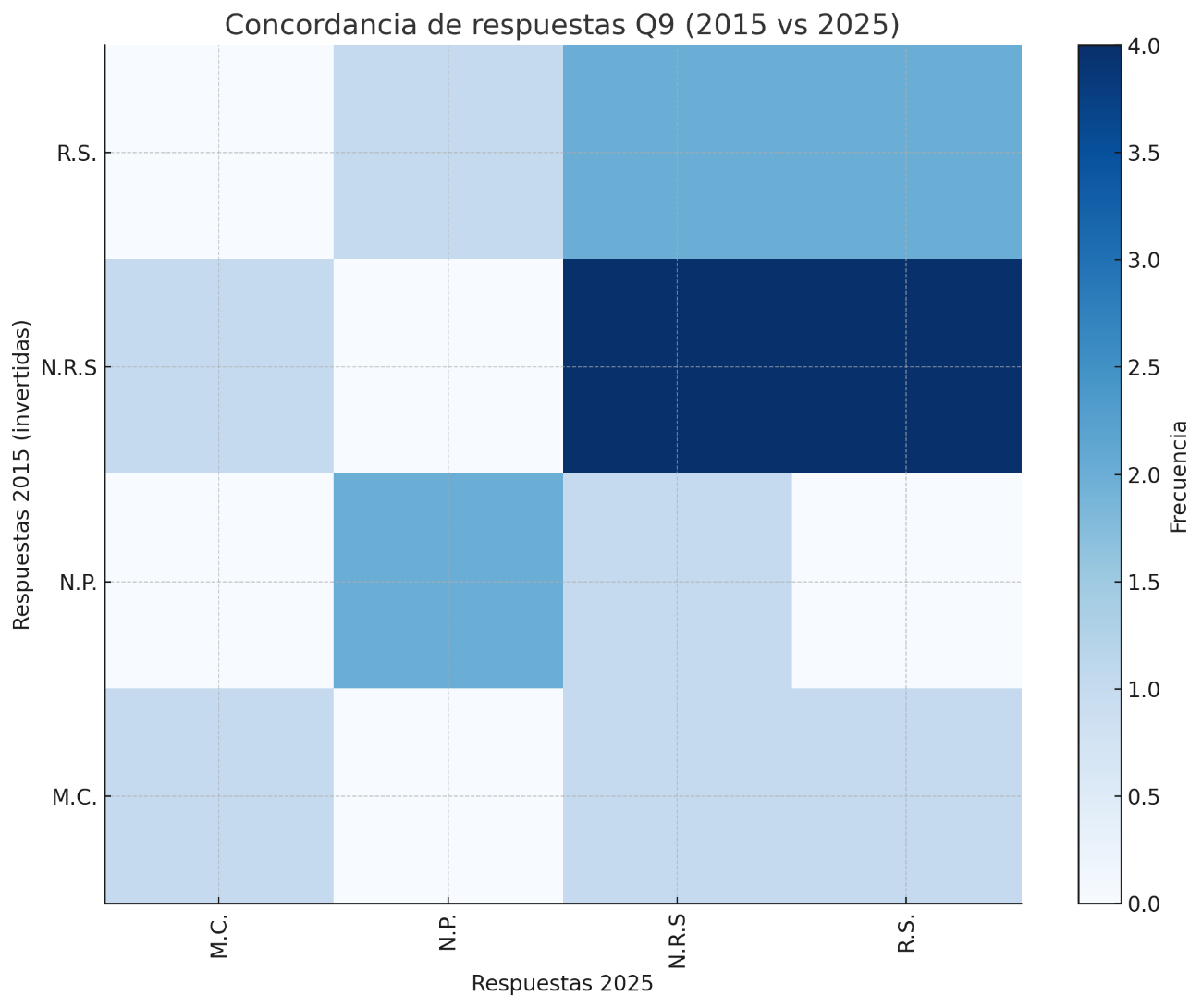


Answers 2025

Frequency

Answers 2015

N.R.S: non-resorbable stiches, R.S: resorbable stiches, M.C.: metallic clips; N.P.: no pockets, I inject.
